# Inflammatory Remission in T2 Severe Asthma

**DOI:** 10.3389/falgy.2022.923083

**Published:** 2022-05-30

**Authors:** Manuel J. Rial, Javier Domínguez-Ortega

**Affiliations:** ^1^Department of Allergy, Complexo Hospitalario Universitario A Coruña, A Coruña, Spain; ^2^Department of Allergy, Hospital Universitario La Paz, Institute for Health Research, Madrid, Spain

**Keywords:** severe asthma, biomarkers, T2 asthma, eosinophils, remission

Asthma is the most common chronic respiratory disease, affecting more than 300 million people worldwide (1). The cornerstone of asthma treatment over the past 50 years has been and continues to be, inhaled and in some circumstances, oral glucocorticoids. Corticosteroids are very effective drugs, but their non-specific mechanism of action, based on anti-inflammatory effect, has not been shown to have a significant long-term impact on the course of the disease (2). According to Menzies-Gow et al. (2), clinical remission of asthma implies 12 months or longer without significant symptoms or the use of corticosteroid medications, as well as improved lung function tests. Asthma remission could be split into three major groups: clinical remission, inflammatory remission, and complete remission (Table 1). Although currently no cure is available, it is widely recognized that some asthmatic patients can spontaneously enter in remission later in life, as in allergy sufferers who cease exposure to the causative agent (3). Longitudinal studies conducted in the adult population show a remission rate of around 20% of subjects over a 10-year period, with moderate variations between studies, with a higher percentage of remission among milder patients (4). In adults under 50 years of age, smoking, allergic sensitization, female sex, older age, and high body mass index (BMI) are risk factors for non-remission or inadequate asthma control, without knowledge of the age of onset of asthma (5). Novel therapies emerged in the last years and new ones soon, offers an opportunity to consider clinical and inflammatory asthma remissions as targets to be achieved. The concept of asthma remission has not had a unanimous consensus in recent years and the different results in longitudinal studies are partly due to the different definitions taken into account (4, 5). Subjects in clinical remission continue to have some degree of lung function impairment or bronchial hyperresponsiveness, whereas in complete asthma remission bronchial hyperresponsiveness would no longer be present. In inflammatory remission, airway or serum biomarkers of inflammation (eosinophils, allergen-specific IgE, periostin, FENO...) would be very low or undetectable, but variability of obstruction and hyperresponsiveness would still be present (6). This remission group is the most easily achievable at present in severe asthma with type 2 (T2) inflammation, biologics targeting key mediators of T2 inflammation, including interleukin (IL)-5, IL-4/IL-13, and immunoglobulin (Ig)E. It should be noted that in all three remission groups there is some degree of airway remodeling, therefore it would not formally be a cure of the disease.

**Table 1:** Types of asthma remission, their definition and implications on lung function.

	**Definition**	**Lung function**
Clinical remission	12 months or longer without significant symptoms or the use of corticosteroid medications, as well as improved lung function tests	Some degree of lung function impairment or bronchial hyperresponsiveness
Inflammatory remission	Airway or serum biomarkers of inflammation (eosinophils, allergen-specific IgE, periostin, FENO...) would be very low or undetectable	Variability of obstruction and hyperresponsiveness would still be present
Complete remission	Absence of asthma symptoms without the use of medication.	Bronchial hyperresponsiveness would no longer be present

The concept of remission usually includes freedom from the need for controller medication. In severe uncontrolled asthma treated with a biologic drug, patients can reduce or even eliminate the need for oral corticosteroids and reduce or even eliminate the need for rescue bronchodilators, but complete elimination of the use of inhalers (ICS/LABA, LAMA) is not common (7). The high price of biologic therapies makes them a therapeutic option in the most severe forms of asthma, where bronchial remodeling is more present and lung function is more impaired. In these situations of lung function deterioration, even if the asthma remains stable, there is a lack of evidence to recommend withdrawal of the controller drug. If currently available biologic therapies, or those yet to come, were to result in the patient not needing any treatment at all, it is possible that health systems would force biologic discontinuation in favor of cheaper treatment options. The use of biologics blocks a specific pathway that in some patients may lead to effective control of inflammation if it is the main pathway involved in that case. In some others, there may be alternative or secondary pathways which, after the blockade induced by the prescribed drug, may be over-activated and may not achieve complete remission.

In real life, biologic drug discontinuation has been performed with omalizumab concluding that it can be performed after prolonged use (>2 years) in children with non-active allergic disease, prolonged controlled asthma and no severe exacerbations for at least 1 year (7). A real-life adult study suggest that the effects of 6 years of omalizumab may persist after discontinuation of therapy in 60% of patients for at least 4 years (8). However, in adults, an increased blood eosinophil count at baseline and a significant increase in FeNO from baseline to week 12 after discontinuation of omalizumab were associated with clinical deterioration (9). The COSMOS trial (10) of 592 patients evaluated changes in ACQ-5 score and blood eosinophil counts 12 weeks after discontinuation of mepolizumab. 12 weeks after the last administration of mepolizumab, the mean ACQ-5 score had increased in parallel with the increase in blood eosinophil counts. The mean increase in ACQ-5 score after discontinuation was only 0.35 points, which is not considered clinically significant. Thus, this study indicated that cessation of mepolizumab does not contribute to a significant deterioration in asthma symptoms during the 12 weeks following discontinuation. It is important to bear in mind that averages are normally used so there are patients in whom the deterioration is important. It should also be noted that there are patients in whom the deterioration in control does not occur so early, opening the door to assessing in which types of patients the responses would be more prolonged. According to these studies, the interruption of biological products may be a feasible strategy in suitable patients with severe asthma, although the predictors of success must be analyzed. In the XPORT study, patients who successfully discontinued omalizumab had lower peripheral eosinophil counts than those who failed to discontinue treatment, indicating that suppressed T2 inflammation may be a predictor of successful discontinuation. In the Vennera study, patients who successfully discontinued tended to have fewer asthmatic comorbidities (e.g., sinusitis, nasal polyps). COMET study concluded that mepolizumab cessation led to deterioration in asthma control. This may simply indicate that residual asthma symptoms are associated with worsening asthma. Further research into predictors of sustained well-controlled conditions after discontinuation of biologics is required to identify patients who are suitable candidates for biologics interruption.

Future research should focus on at least two aspects: determining true remission from a histological point of view and performing more thorough analyses of the biological pathways to explore the triggers that cause this phenomenon. The remission of asthma as a treatment objective, allows to advance in its treatment and improve the results obtained, similar to what has been achieved in other chronic inflammatory diseases.

## Author Contributions

All authors listed have made a substantial, direct, and intellectual contribution to the work and approved it for publication.

## Conflict of Interest

The authors declare that the research was conducted in the absence of any commercial or financial relationships that could be construed as a potential conflict of interest.

## Publisher's Note

All claims expressed in this article are solely those of the authors and do not necessarily represent those of their affiliated organizations, or those of the publisher, the editors and the reviewers. Any product that may be evaluated in this article, or claim that may be made by its manufacturer, is not guaranteed or endorsed by the publisher.
